# Evaluation of hearing level in patients on long term aspirin therapy

**DOI:** 10.12688/f1000research.11131.2

**Published:** 2018-02-16

**Authors:** Apar Pokharel, Sangita Bhandary

**Affiliations:** 1 ENT Department, College of Medical Sciences, Bharatpur, Chitwan, Nepal

**Keywords:** aspirin, sensorineural hearing loss, cardiovascular diseases

## Abstract

**Introduction**: Aspirin is a routinely prescribed drug, most notably for cardiovascular diseases, such as myocardial ischemia. This cross sectional, comparative study study aims to explore differences in hearing status between the cardiovascular disease patients on aspirin therapy and age matched controls.

**Methods**: The study population consisted of 182 patients with heart disease taking long term aspirin (i.e., for more than one year). The control population consisted of 221 age matched controls who were not taking aspirin.

**Results**: It was found that age of patient, not aspirin intake, was more important risk factor contributing to hearing loss.

**Conclusions**: When confounding factors like age of the patient, hypertension and diabetes were taken into account, aspirin in its antiplatelet dose was not found to be the cause of any audiological problems like tinnitus and hearing loss.

## Introduction

The great industrial and technological revolutions of the past two centuries have resulted in changes in the causes of illnesses and death. Infections and malnutrition were the most common cause of death before 1900. Because of improved nutrition and public health measures in the developed countries, the greatest cause of morbidity has been cardiovascular disease (CVD) and cancer. As lifestyle changes have also been observed in developing countries, morbidity and mortality rates due to cardiovascular conditions are also becoming more common in these countries. This is known as the epidemiological transition because this shift is highly correlated with changes in personal and collective wealth (the economic transition), social structure (the social transition) and demographics (the demographic transition) ^1^.

Aspirin is a routinely prescribed drugs, most notably for cardiovascular diseases such as, myocardial ischemia. Myocardial ischemia or ischemic heart disease (IHD) is a disease where there is a decreased blood supply to the heart muscle due to coronary artery disease (CAD) ^2^. The ototoxic effects of high doses (several grams per day) of salicylates, reversible hearing loss and tinnitus, are well documented ^3^.

No studies have been done in the past to assess hearing loss in patients with cardiovascular diseases on aspirin therapy in Nepal. The present study was done to explore the differences in hearing status between patients with cardiovascular disease on aspirin therapy and age matched controls. It also aims to analyze correlation of hearing loss with the age of patients and presence of co-morbid illnesses.

## Methods

The study was conducted in the department of Otorhinolaryngology and Head & Neck Surgery, BPKIHS, Dharan. The duration of the study was one year (from 1 ^st^ Feb, 2011 to 1 ^st^ Feb, 2012). This was a cross sectional comparative study.

The study population consisted of cases of patients with heart disease taking long term aspirin (i.e., for more than one year). They were informed about the design and purpose of the study and requested to visit the ear, nose and throat outpatient department (ENT OPD) voluntarily to take part in the study. The control population consisted of age matched controls who were not taking aspirin. No major, active interventions were carried out other than those routinely required for diagnosis. Ethical approval was obtained from the institutional ethical review board of B.P. Koirala Institute of Health Sciences (87/2070/071). Written informed consent was obtained from both study and control populations prior to the study. The current study did not involve any invasive procedure and did not cause any physical or mental harm to the patients. No other interventions were carried out. There was no financial burden to the patients during study. The inclusion criteria was patients of age group 15–75 years who were taking aspirin for heart disease. The exclusion criteria was patients with hearing loss after trauma i.e. after explosion, head injury, ear trauma, or perforation of tympanic membrane, and patients with positive family history of hearing loss, or with CNS disease.

All study participants visited ENT OPD so that their clinical history could be assessed according to the proforma and so that they could undergo clinical and otological examination. General physical examination and detailed otological examination of ear, nose and throat was carried out according to proforma to exclude middle ear pathology and conductive hearing loss. All the study participants had normal ears during ear examinations. The Heine Mini 3000 otoscope was used in all cases for the examination of the external auditory canal and the tympanic membrane.

The study population was asked about regular use of aspirin which was defined as daily intake of 75mg ^4^. The study population was interviewed regarding hearing loss and tinnitus and any past history of ear disease was excluded. Tinnitus is a recurrent ringing, roaring or buzzing sensation lasting for five minutes or longer ^5^.

Complete birth and developmental history was taken to exclude congenital and other causes of acquired hearing loss. Detailed drug history was taken, looking especially for ototoxic drugs. Past history of ear trauma and head injury was noted to rule out prior hearing loss. Systemic causes of hearing loss e.g. Diabetes mellitus, hypertension, and chronic kidney disease, should also be noted. The diagnosis of diabetes mellitus, hypertension and chronic kidney disease were made based on the previous medical records of study population and controls.

Tuning fork tests (Rinne’s, Weber’s) were carried out with 256, 512, 1024 Hz tuning fork. Lower frequency fork e.g. 128 was not used because of difficulty in interpretation. Rinne and Weber tests were done to find the better hearing cochlea. Each participant underwent a hearing test by pure tone audiometry (PTA) in a sound-proof room. The Madsen Electronics Orbiter 922 Version 2 clinical audiometer was chosen for the study, and tests were performed by a trained audiometrician. The audiometric testing was done by a single person to ensure test-retest reliability. The extent of hearing loss was determined in all subjects. An average of two pure tone responses was calculated in cases of doubt. The results were documented as low (250–500 Hz), middle (1000–2000KHz), and high (4000–8000Hz). The value of the worst ear was taken when there was slight difference in the hearing loss value between the two ears. Hearing thresholds of the study group were compared with those of control group. All findings were noted and categorized on the basis of a proforma data collection sheet. A graph was plotted with the PTA findings. The data were entered in Excel and analysis was carried out using SPSS version 16.0. The Chi-squared test was done to compare hearing loss between case and control, across different age groups and at different durations of intake of aspirin. 

## Results

The study population consisted of 182 patients with heart disease taking long term aspirin (i.e., for more than one year). The control population consisted of 221 age matched healthy controls who were not taking aspirin. 45.3% of the study population were male and 54.7% were female. Among controls, 48.1% of the cases were male and 51.9% of the cases were female ( Table 1).

**Table 1.** Demographic Data.

		Study Population	Control Population
**Age**	**Less than 50 yrs**	20.9%	21.7%
	**50–59 yrs**	31.3%	29.7%
	**60–75 yrs**	47.6%	48.4%
**Sex**	**Male**	45.3%	48.1%
	**Female**	54.7%	51.9%
**Race**	**Indo-Aryans**	64.3%	60.1%
	**East-Asians**	35.7%	39.9%

Both study and control populations were categorized into three groups according to age. The first group ranged between 15–50 years of age, the second 51–59 and the third, 60–75. The mean age was 63.7 years for the study population and 64.2 years for controls. 47.6% of the study population was in the 60–75 year age group; in the control group the number of participants in the 60–75 age group which was higher, with 48.4% ( Table 1).

64.3% of the study population were of Indo-Aryan origin and 35.7% were of East Asian origin. Among the controls, 60.1% were of Indo-Aryan origin and 39.9% were of East Asian origin ( Table 1).

**Figure 1.**
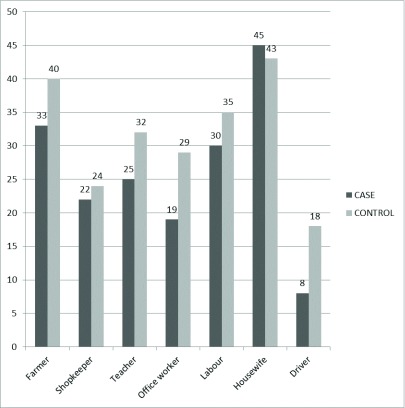
Occupation distribution between cases and controls (182 cases and 221 controls).

The occupation of participants was more or less equally distributed, with housewives dominating in both cases and controls. Careful history was taken to rule out noise induced hearing loss in each occupation as seen in Figure 1.

**Figure 2.**
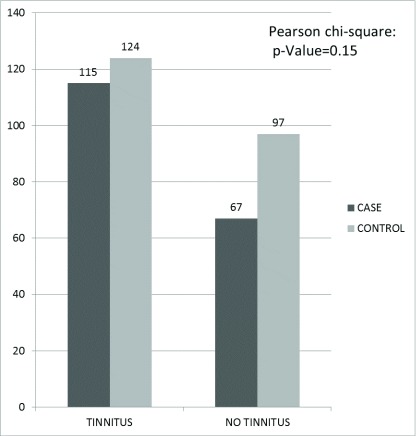
Incidence of tinnitus among cases and controls (182 cases and 221 controls).

Out of 182 study population, only 115 presented with tinnitus. Among 221 controls, 124 presented with tinnitus. The result was statistically insignificant as in Figure 2.

**Figure 3.**
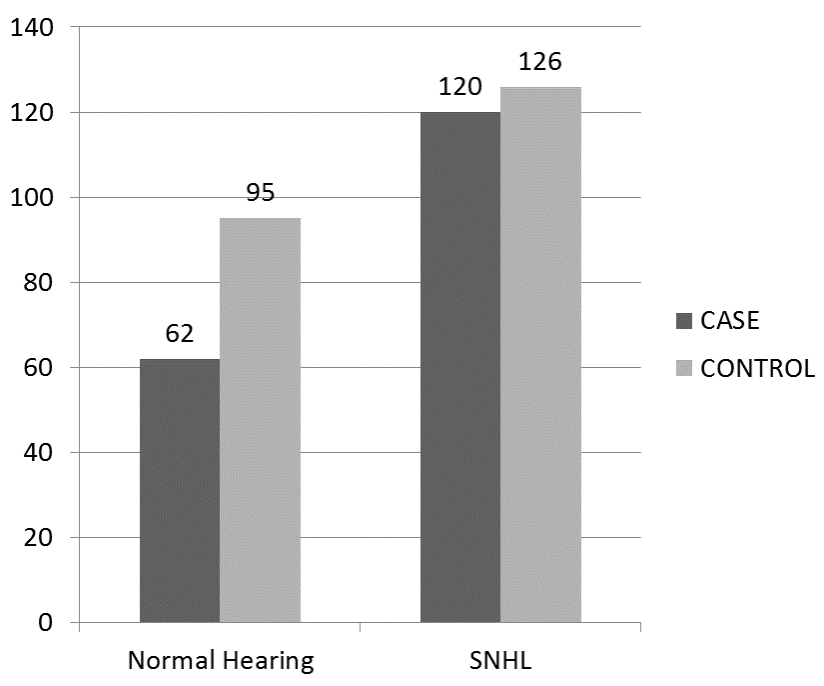
Incidence of sensorineural hearing loss among cases and controls (182 cases and 221 controls).

Out of 182 study population, only 120 presented with sensorineural hearing loss (SNHL). Among 221 controls, 126 presented with SNHL. The result was not statistically significant (p value= 0.68) implying that aspirin was not the cause of hearing loss in study population as seen in Figure 3.

**Figure 4.**
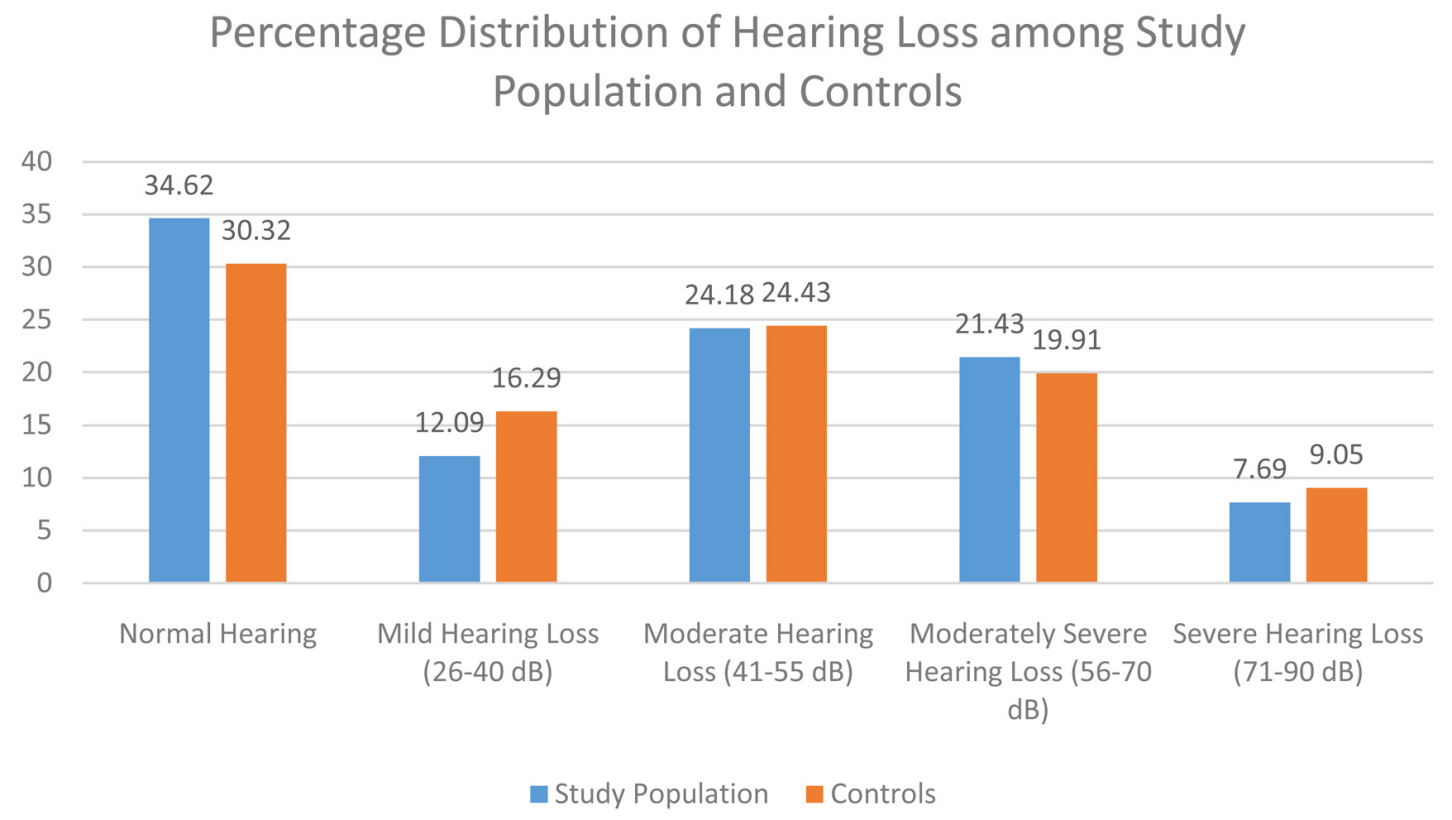
Percentage Distribution of Hearing Loss Among Study Population and Controls.

As seen in Figure 4, maximum percentage of both the study population and controls have normal hearing. Around 24% of both study population and controls have moderate hearing loss as per WHO criteria.

The data obtained from bivariate analysis was followed by binary logistic regression analysis with backward conditional method using SPSS version 16.0 in order to adjust and explore the significance of explanatory variables. Hearing loss was considered the dependent variable. The independent variables which had p value of 20% or less in the bivariate analysis were then included for binary logistic regression analysis. The variables subjected to the model were age group, duration of aspirin use, hypertension and diabetes mellitus.

When logistic regression was applied among the study population it was found that age of the patient was most significantly associated with hearing loss. Patients aged 51–59 were 14.258 times more prone to develop hearing loss and patient aged 60–75 were 61.389 times more prone to develop hearing loss than patients aged less than 50 years of age ( Table 2).

 It was also found that only age of the study population was significantly associated with tinnitus. Duration of aspirin use, hypertension and diabetes were not found to be associated with tinnitus. People less than 50 years of age were 32.612 less likely to develop tinnitus than people between 60–75 years of age. Similarly patients between 51–59 years of age were 2.799 times less likely to develop tinnitus than people aged between 60–75 years ( Table 3).

**Table 2.** Regression analysis of age, duration of aspirin intake, hypertension (HTN) and diabetes (DM) with hearing loss (182 cases).

	PARAMETERS	STANDARD ERROR	SIGNIFICANCE	RISK RATIO (EXP B)	95% CONFIDENCE INTERVAL FOR EXP(B)	
					LOWER BORDER	UPPER BORDER
**Step 1**	Age(<50yrs)		<0.001			
	Age(51–59yrs)	1.113	0.026	11.828	1.336	104.743
	Age(60–75yrs)	1.105	<0.001	49.277	5.649	429.860
	Duration of aspirin (1–4yrs)		0.285			
	Duration of aspirin (5–8yrs)	0.388	0.212	1.624	0.759	3.473
	Duration of aspirin (>8yrs)	0.690	0.176	2.544	0.658	9.843
	HTN(Yes)	0.541	0.630	1.297	0.449	3.745
	DM(Yes)	0.380	0.390	1.386	0.659	2.917
**Step 2**	Age(<50yrs)		<0.001			
	Age(51–59yrs)	1.079	0.016	13.531	1.634	112.08
	Age(60–75yrs)	1.080	<0.001	55.477	6.686	460.299
	Duration of aspirin (1–4yrs)		0.29			
	Duration of aspirin (5–8yrs)	0.387	0.203	1.636	0.766	3.494
	Duration of aspirin (>8yrs)	0.688	0.187	2.478	0.643	9.547
	DM(Yes)	0.359	0.453	1.310	0.648	2.648
**Step 3**	Age(<50yrs)		<0.001			
	Age(51–59yrs)	1.077	0.013	14.379	1.741	118.783
	Age(60–75yrs)	1.079	<0.001	58.811	7.101	487.104
	Duration of aspirin (1–4yrs)		0.256			
	Duration of aspirin (5–8yrs)	0.386	0.184	1.67	0.784	3.556
	Duration of aspirin (>8yrs)	0.682	0.167	2.57	0.675	9.789
**Step 4**	Age(<50yrs)		0.013			
	Age(51–59yrs)	1.067	<0.001	14.258	1.761	115.434
	Age(60–75yrs)	1.070	0.013	61.389	7.543	499.591

**Table 3.** Regression analysis of age, duration of aspirin intake, hypertension (HTN) and diabetes (DM) with tinnitus (182 cases).

PARAMETERS	STANDARD ERROR	SIGNIFICANCE	RISK RATIO EXP (B)	95% CONFIDENCE INTERVAL FOR EXP(B)	
				LOWER BORDER	UPPER BORDER
Age(<=50 YRS)	0.826	<0.001	32.612	6.465	164.499
Age(51–59 YRS)	0.357	0.004	2.799	1.390	5.638
Age(60–75 YRS)					
HTN(ABSENT)	0.682	0.409	0.644	0.226	1.831
HTN(PRESENT)					
DM(ABSENT)	0.081	0.776	1.107	0.549	2.232
DM(PRESENT)					
DURATION OF ASPIRIN(1–4 YRS)	0.257	0.612	1.361	0.413	4.481
DURATION OF ASPIRIN(5–8 YRS)	0.014	0.907	0.936	0.306	2.860
DURATION OF ASPIRIN(>8YRS)					

Raw data collected from the study population Click here for additional data file. Copyright: © 2018 Pokharel A and Bhandary S 2018 Data associated with the article are available under the terms of the Creative Commons Zero "No rights reserved" data waiver (CC0 1.0 Public domain dedication).

Raw data collected from the control population Click here for additional data file. Copyright: © 2018 Pokharel A and Bhandary S 2018 Data associated with the article are available under the terms of the Creative Commons Zero "No rights reserved" data waiver (CC0 1.0 Public domain dedication).

## Discussion

In the present study, the mean age of the patient was 63.7 years. As most patients on regular aspirin were above the age of 60, aspirin was not seen as the principal cause of sensorineural hearing loss in our study. Logistic regression analysis demonstrated that- other factors like duration of aspirin intake, and other comorbidities like hypertension and diabetes were not found to produce any significant effect on the hearing status of the patient. Our results oppose Curhan *et al*’s, which had shown that aspirin given for its anti-platelet effect in cardiovascular patients is responsible for causing hearing loss even in low doses. Hearing loss was seen as one of the complications of regular analgesic use. The risk was seen greatest among men below the age of 60 years. Above the age of 60 years, no relation was seen between hearing loss and aspirin use ^4^.

With increasing age, the prevalence of the hearing loss also increases ^6^. Studies have shown that after the age of 60, hearing thresholds worsen on average by 1 dB per year, but the rate of decline of hearing loss may be even greater in men aged 48–59 years ^7, 8^. The relationship between regular use of aspirin and hearing loss was observed as strongest in men younger than 60 years of age. A possible explanation for this might be that after the age of 60 the cumulative effects of age and other comorbidities will add up in the causation of hearing loss. Bainbridge *et al* showed similar impact of age and diabetes on hearing loss ^9^.

High doses of salicylates can have ototoxic effects which include reversible hearing loss and tinnitus. Animal models have shown that salicylate is responsible for abnormal function of the outer hair cell and decreased blood flow in the cochlea ^3^. Membrane conductance of the outer hair cell changes because of biochemical and electrophysiological alteration induced by salicylates. Salicylates also cause auditory microvasculature vasoconstriction, most probably caused by their antiprostaglandin activity ^10, 11^.

Histopathologic studies of human temporal bones and animals on salicylate administration show degeneration of the strial microvasculature ^12, 13^. Microvascular vasoconstriction leads to strial degeneration. However, strial vascularis degeneration is also one of the characteristic features of age-related hearing loss ^14^. Animal studies have shown that strial degeneration leads to its atrophy and to capillary loss, with basement membrane thickening and deposition of laminin and immunoglobulin in the strial vasculature ^15– 17^.

 Inflammatory mediators like TNF-α affects microvascular tone, thereby reducing cochlear blood flow ^18^. Other inflammatory biomarkers like white blood cell count, neutrophil count, IL-6 and CRP are associated with hearing loss in older people ^19^. Over a 10 year period, older people with higher levels of CRP were two times more likely to develop hearing loss than normal ^20^. Aspirin inhibits platelet enzyme cyclo-oxygenase (COX). Increased expression of genes for IL- 1β, IL-6, TNF-α, and COX-2 occurs during prostaglandin biosynthesis, and this effect is inhibited by aspirin ^21^. Aspirin is also responsible for the synthesis of anti-inflammatory compounds called ‘aspirin-triggered 15-epi-lipoxins’. This is responsible for the drug's anti-inflammatory effect even at low doses ^22^. Aspirin also decreases chronic inflammatory biomarkers responsible for the aging process ^23^. A randomized controlled trial where 100 mg of aspirin was given for acute coronary syndrome showed decrease in the level of both CRP and TNF-α ^24^. These studies show that that aspirin in low doses may have a protective effect on hearing instead of a deteriorating effect. Recently, a clinical trial is going on to see whether aspirin in low doses can decrease the progression of age-related hearing loss ^25^.

This study has certain limitations. Aspirin in low dose is an over the counter medication and is used by a large proportion of the population. To find out whether aspirin is responsible for causing hearing loss or not, sample size needs to be huge, and the samples should be followed up for a long period of time. Because of time constraints, this was not possible for this study. Hearing evaluation before the patient began aspirin treatment was also not carried out. The lowest age group patient in this study was 34 years and patients below 50 years of age were very few in number so the first age group made was from 15–50 years.

## Conclusions

Hearing impairment is an important public health issue. Aspirin is one of the most common medications used now-a-days owing to the increased prevalence of cardiovascular diseases. The present study shows that long term use of aspirin doesn’t cause any hearing loss. The relationship between long term use of low dose aspirin and hearing loss is still a debatable subject. Other factors like age of the patient, hypertension and diabetes mellitus must also be looked at if a person on aspirin develops symptoms of hearing loss.

## Data availability

The data referenced by this article are under copyright with the following copyright statement: Copyright: © 2018 Pokharel A and Bhandary S

Data associated with the article are available under the terms of the Creative Commons Zero "No rights reserved" data waiver (CC0 1.0 Public domain dedication). 

**Dataset 1: Raw data collected from the study population.**

DOI, 10.5256/f1000research.11131.d156960 ^26^

**Dataset 2: Raw data collected from the control population.**

DOI, 10.5256/f1000research.11131.d156961 ^27^

## Supplementary material

Supplementary File 1: A table showing air conduction and bone conduction threshold of study population across different frequencies.

Supplementary File 2: A table showing air conduction and bone conduction threshold of controls across different frequencies.
