# Supplementary figures and images for: Evaluation of hearing level in patients on long term aspirin therapy
Source: F1000Res. 2018 Feb 16;6:445. Originally published 2017 Apr 7. [Version 2] doi: 10.12688/f1000research.11131.2 (PMC5909044; doi:10.12688/f1000research.11131.2)

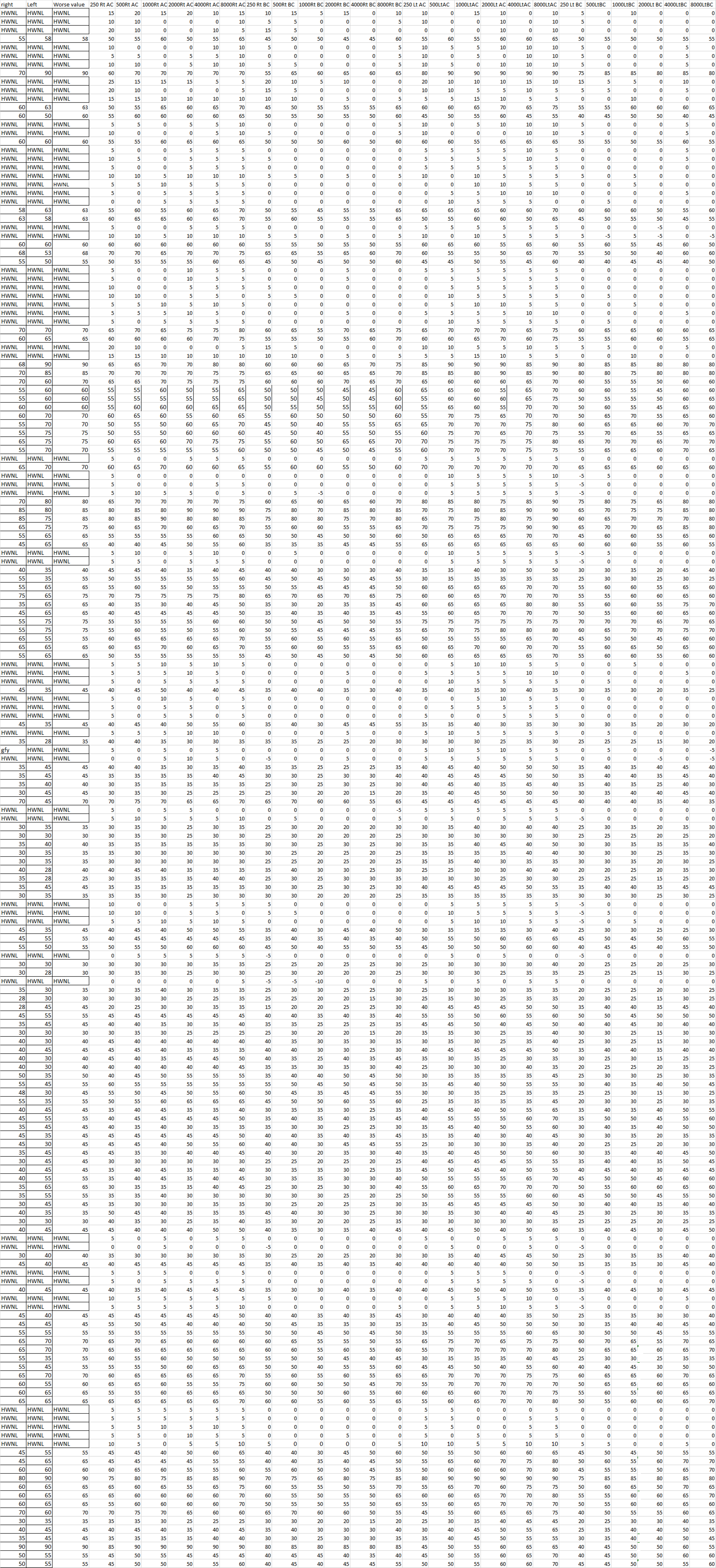

Supplement: Supplementary file 3 [file f1000research-6-15247-s0002.tgz › 98a4e339-e4db-4800-9fda-f72875f3c282.png]

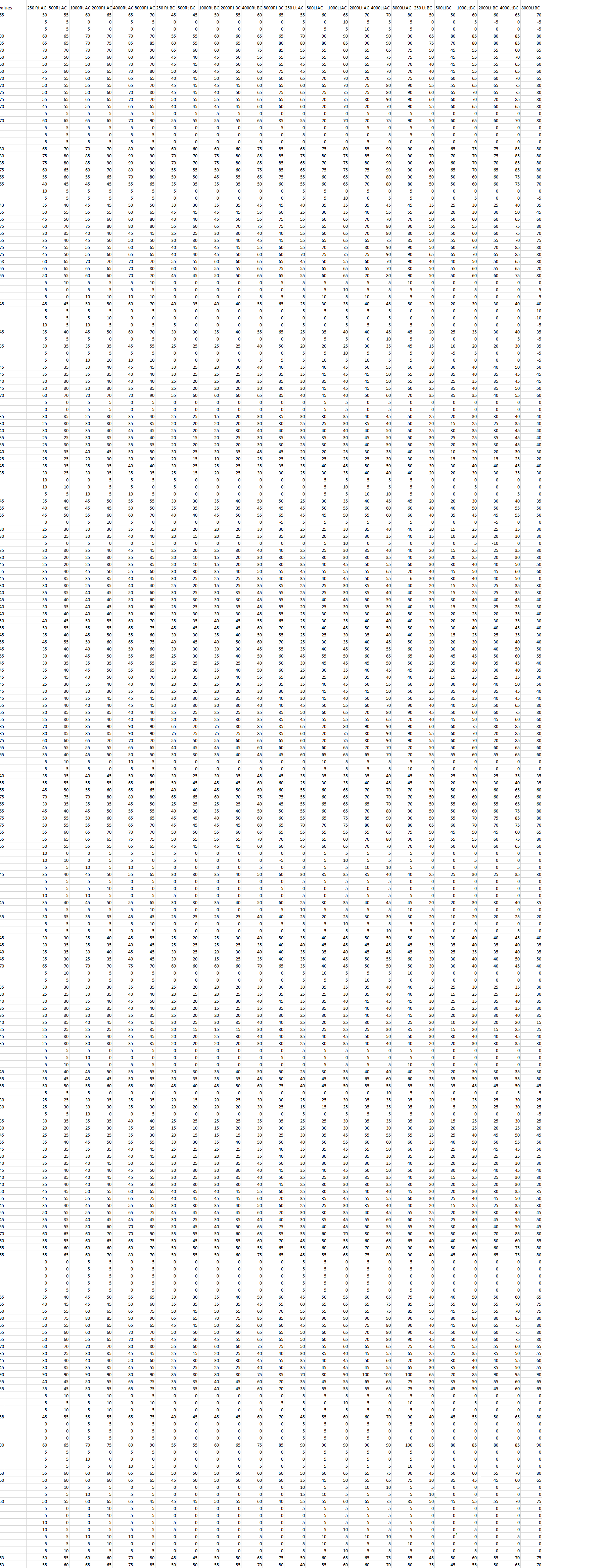

Supplement: Supplementary file 4 [file f1000research-6-15247-s0003.tgz › 3995377d-3fe8-4bfa-b5ef-535669a9bcfd.png]
