# Supplementary material for: Evaluation of hearing level in patients on long term aspirin therapy
Source: F1000Res. 2018 Feb 16;6:445. Originally published 2017 Apr 7. [Version 2] doi: 10.12688/f1000research.11131.2 (PMC5909044; doi:10.12688/f1000research.11131.2)
Supplement: Peer review history

# Peer review: Referee response for version 2

**DOI:** 10.5256/f1000research.15247.r30913

**Published:** 2018-04-18

**Authors:** Elsdon Storey

**Affiliations:** 1 Department of Medicine (Neuroscience), Monash University, Melbourne, Vic, Australia

I have read this submission. I believe that I have an appropriate level of expertise to confirm that it is of an acceptable scientific standard.

# Peer review: Referee response for version 1

**DOI:** 10.5256/f1000research.12008.r21660

**Published:** 2017-05-19

**Authors:** Ajoy Mathew Varghese, Sheeja Susan John

**Affiliations:** 1 Department of Otolaryngology and Head and Neck Surgery, Christian Medical College, Vellore, India; 2 Department of Ophthalmology, Christian Medical College, Vellore, India

**Introduction**

1. Research question: The authors have aimed to look at the effect of prolonged use of low dose aspirin on hearing. The question is relevant as most of the previous studies on the subject have looked at the effect of high dose aspirin.
2. Aim and objectives stated in abstract and introduction differ from the ones stated in the Methods section. These need to be stated clearly and consistently.

 

**Methods**

  

1. Study design: ****The authors have done a cross sectional comparative study between patients with cardiac disease on aspirin, and controls without cardiac disease and not on aspirin.
2. Controls cannot be termed ‘healthy’. They were age-matched subjects without heart disease, and not on aspirin therapy.
3. If age matched controls were selected, why is there a difference in the number of cases and controls? Were there any drop outs from the study?
4. What were the criteria for diagnosing hearing loss? Since patients did not have a baseline audiological test, how were hearing loss and progression of hearing loss defined? Were questionnaires used? Where they standardized?
5. Systemic risk factors for hearing loss e.g. Diabetes mellitus, hypertension, and chronic kidney disease, also had to be ruled out.’ (para 5 in Methods section) These factors were present both in cases and controls (see Datasets). These factors were not ruled out. They were noted as potential confounders in both groups, and addressed in the logistic regression analysis. 
6. Chronic kidney disease has been mentioned along with diabetes and hypertension as a confounding factor in the beginning of the manuscript. However, there is no mention of chronic kidney disease in the rest of the manuscript and data sets, and it has not been addressed in the logistic regression model. Please clarify.

 

**Results**

  

1. Baseline demographic data should be put in a table to ensure clarity.
2. Please depict the tinnitus and hearing loss in cases and controls, with p-values, as tables.
3. CKD was not included as part of the logistic regression. Why not? Please clarify. The textual interpretation of this could also be better worded.
4. Audiometric data need to be added both in the data set and the results.

 

**Discussion**

  

1. The discussion needs to be clearer, more concise, and should proceed in a logical sequence. In the discussion, the authors should cite studies that have shown that aspirin could cause hearing loss, and how the current study differs from them, and possible reasons as to why. Then they should discuss studies and theories to the contrary, that agree with the results of the current study. This should be done in a logical and concise manner. Please avoid unnecessary and confusing details of various theories that have no direct implication on the subject of research.
2. The ‘prevalence’ of hearing loss in both cases and controls is far above the prevalence quoted in existing literature. Is there an explanation for this?
3. Please give reference number for Curhan *et al’s *paper at the end of the first citation in the manuscript. (para 1 in discussion)
4. The comparison of the present study with Curhan *et al’*s work is not very clear. Are the authors trying to imply that their results conflict with those of this work because the majority of their study population (both cases and controls) was above the age of 60? Please make this clear.

 

**Conclusion**

  

1. Conclusion should correspond with the aim and objectives of the study. The present study found no relationship between long term, low dose aspirin use and hearing loss. Moreover, the study did not find any correlation between hearing loss and systemic diseases; hence the last line in the conclusion is not supported by the results of the study, and therefore, may be deleted.

 

**General comments**

  

1. Correction of grammar needs to be done throughout the manuscript.
2. Terminology such as ‘cases’ and ‘controls,’ and ‘incidence’ and ‘prevalence’ has been used loosely and interchangeably. This is not acceptable. 

 

 On the whole, the paper addresses a relevant research question, and may be indexed after making the suggested corrections.

We have read this submission. We believe that we have an appropriate level of expertise to confirm that it is of an acceptable scientific standard, however we have significant reservations, as outlined above.

# Peer review: Referee response for version 1

**DOI:** 10.5256/f1000research.12008.r22554

**Published:** 2017-05-08

**Authors:** Elsdon Storey

**Affiliations:** 1 Department of Medicine (Neuroscience), Monash University, Melbourne, Vic, Australia

**Introduction:**

 It is well-known that large doses of aspirin may produce reversible tinnitus and hearing loss.  The authors set out to test whether prolonged use of low dose aspirin for heart disease (presumably mostly ischaemic in nature) has a deleterious effect on hearing.  Unfortunately, there are a number of problems with this study.

**Subjects:**

1. The authors are correct in their acknowledgement of vascular risk factors such as hypertension and diabetes for age-related hearing loss.  (This age-related loss is presumably predominant in this study, judging from the increase in deafness by age group.)  The design of the study does not allow ischaemic heart disease – itself also a risk factor for age-related hearing loss – to be accounted for, as all the aspirin-taking group were on aspirin for heart disease, whereas none of the control group had this risk factor.  This confounding factor can only be addressed in a blinded, randomised prospective trial of aspirin in healthy individuals, as the authors acknowledge.
2. There is some confusion regarding the risk factors diabetes and hypertension in the manuscript.
  a) What criteria were used for these diagnoses?  Furthermore, were the subjects screened for these risk factors as part of the study, or was there reliance on medical record review or on patient self-description?
  b) These risk factors “ ... had to be ruled out” (Methods, para 5, page 2), but these factors were included in the regression analysis (Results, para 7, page 3).  Were those with hypertension and diabetes indeed excluded from the study?
3. While there was adjustment for age and sex, other known risk factors for age-related hearing loss such as smoking and educational level (Stevens G, *et al.* 2013 ** ^1^; Agrawal Y, *et al.*  2008 ^2^) were not reported.  Were there any group differences in these factors?
4. The age range stratification (15-50; 51-59; 60-75) seem rather arbitrary and uneven.  Were these divisions chosen to equalise numbers?  The grouping of 15 year olds with 50 year olds seems inappropriate, and presumably the heart disease for which the 15 year olds were on aspirin was rather different.  I think that the study would have been stronger with the exclusion of this paediatric group (presumably small).  A histogram of the age distribution of subjects and controls by year of age (not age group) would be illuminating.

**Methods:**

 Figure 1:  The definition of hearing impairment is unclear.  Why was the WHO classification not used?  This averages the loss in dB at 4 frequencies:  0.5, 1, 2 and 4 kHz in the *better* ear, with mild loss being diagnosed at > 25 dB and moderate at > 40 dB.  I presume that a dichotomous classification of hearing impairment was applied given that chi-squared tests were applied to the data, but the definition of hearing loss is not actually stated.

 In a related matter, the results were documented as low, middle, high frequency loss (Methods, para 6, page 2), but this stratification is not subsequently referred to in the Results section.

 Noise-related deafness was excluded on history (“careful history was taken to rule out noise-induced hearing loss in each occupation” – Results, para 4, page 3).  Was the presence of a prominent notch on PTA at 4 kHz used to complement the history of noise exposure as a cause of exclusionary noise-related deafness?  (Incidentally, this exclusionary statement should appear in Methods rather than Results.)

 Conductive loss was excluded on clinical examination and tuning fork tests.  Was there a reason that a bone-ear conduction gap on audiometry was not used to complement this exclusionary point?

**Results:**

 The results are tabulated with inappropriate precision (often to 4 or 5 significant figures). 

**Discussion:**

 Age-related hearing impairment is presumably the major cause of deafness in this study, as the relative risks of the older age bands suggest.  There is animal evidence for microvascular involvement in age-related hearing loss (Prazma J, *et al*. 1990 ^3^; Shi X. 2011 ^4^).  There is evidence from humans linking inflammatory states to age-related hearing loss by correlation of markers of mild systemic inflammatory states (raised IL-6 and CRP levels) with hearing loss (Verschuur C, *et al*. 2012 ^5^).  In other words, a reasonable hypothesis would be that low dose aspirin is actually protective against age-related hearing loss.  In this context the study by Curhan *et al.* cited by the authors deserves further discussion.  It found greater rates of hearing loss with prolonged regular use of aspirin, NSAIDs, and paracetamol (acetaminophen).  There are two problems with that study, however:  firstly, it relied on self-report of symptoms of deafness, with confirmation by a professional again self-reported.  A second and potentially greater problem is confounding by indication, in that the reason for taking these medications in the first place (e.g.  mild inflammatory state) may itself have been responsible for some or all of the hearing impairment.

**Conclusion:**

 As it stands, this submission requires considerably more work to reach a suitable level.  However, the primary data is presumably still available, and the submission could be re-written and re-analysed, perhaps along the lines suggested above, to improve its suitability. 

 A minor comment is that the English expression requires some revision, although it is largely understandable in its current form.

I have read this submission. I believe that I have an appropriate level of expertise to confirm that it is of an acceptable scientific standard, however I have significant reservations, as outlined above.

## References

[1] StevensGFlaxmanSBrunskillEMascarenhasMMathersCDFinucaneMGlobal Burden of Disease Hearing Loss Expert Group: Global and regional hearing impairment prevalence: an analysis of 42 studies in 29 countries. *Eur J Public Health*.2013;23(1) : 10.1093/eurpub/ckr176 146-52 10.1093/eurpub/ckr176 22197756

[2] AgrawalYPlatzEANiparkoJK: Prevalence of hearing loss and differences by demographic characteristics among US adults: data from the National Health and Nutrition Examination Survey, 1999-2004. *Arch Intern Med*.2008;168(14) : 10.1001/archinte.168.14.1522 1522-30 10.1001/archinte.168.14.1522 18663164

[3] PrazmaJCarrascoVNButlerBWatersGAndersonTPillsburyHC: Cochlear microcirculation in young and old gerbils. *Arch Otolaryngol Head Neck Surg*.1990;116(8) :932-6 237872110.1001/archotol.1990.01870080054015

[4] ShiX: Physiopathology of the cochlear microcirculation. *Hear Res*.2011;282(1-2) : 10.1016/j.heares.2011.08.006 10-24 10.1016/j.heares.2011.08.006 21875658PMC3608480

[5] VerschuurCADowellASyddallHENtaniGSimmondsSJBaylisDGaleCRWalshBCooperCLordJMSayerAA: Markers of inflammatory status are associated with hearing threshold in older people: findings from the Hertfordshire Ageing Study. *Age Ageing*.2012;41(1) : 10.1093/ageing/afr140 92-7 10.1093/ageing/afr140 22086966

# Response

**Published:** 2017-05-13

**Authors:** Apar Pokharel

**Affiliations:** College of Medical Sciences, Nepal

Thank you for your review. There are few queries raised which I want to address.

1. The patients were all taken from the Department of Internal Medicine. There all patients are screened for blood sugar level and bood pressure. It is very difficult to find pateints with heart disease and  having neither hypertension nor diabetes. So there were included in the study and the risk of hearing loss was calculated using regression analysis.
2. Smoking was taken into account for the hearing loss. To minimize its effect as a confounding factor, in the control group population, smokers were also included.  Education level was not seen in the study as the range of age was very high i.e., 15-65 years.
3. The age grouping was done as 15-50yrs, 51-59yrs, 60-75 yrs because, it is very difficult to get cases with heart diseases on aspirin therapy in age less than 50 years. This age classification is also similar to article by Sharon G. Curhan, MD called  “Analgesic Use and the Risk of Hearing Loss in Men “ published in Am J Med. 2010 March ; 123(3): 231–237.
4. Noise induced Hearing Loss was excluded from history taking as well as from pure tone audiogram findings.

# Peer review: Referee response for version 1

**DOI:** 10.5256/f1000research.12008.r21658

**Published:** 2017-04-19

**Authors:** Adam M. Sheppard

**Affiliations:** 1 Center for Hearing and Deafness, State University of New York at Buffalo, Buffalo, NY, USA

There are typographic errors included a repeated word three lines into the abstract.

 The author’s state that pure tone audiometric evaluations were performed but do not include graphical data in the manuscript, nor do they indicate frequency specific thresholds in the raw data attached, only PTAs. Air conduction and bone conduction information needs to be included in order to ascertain the impact of standing hearing loss has on the included participants, especially since many occupations of participants included are readily exposed to noise such as farmers, labourers, and drivers. Furthermore, ototoxic events primarily initiate at ultra-high frequencies > 8 kHz, which was not even measured in the present study and can have significant impact on conclusions.

 The use of tuning fork is not exactly clear if pure tone audiometry was performed. Unless pure tone audiometry was only performed on the “better hearing cochlea”, in which case the authors should have performed these studies binaurally.

 The studies included do not fully address the impact that salicylate may have on hearing capability. Animal models have shown that salicylate can impact not only DPOAEs but permanently damage spiral ganglion neurons as well. Which could result in recoverable DPOAE’s and thresholds but have a permanent impact on word recognition capabilities. While these observations have been made with significantly higher dosage of salicylate, and may not be present at lower dosages, details should still be included in the manuscript.

 There are many claims in the text which are written as though supported by research. While I believe the text included in the manuscript, proper references need to be included for readers. This event is widespread throughout the manuscript.

 The authors claim their data opposes Curhan *et al’*s research. While findings were different, they need to include the significant differences between the two studies, one primary difference being the number of participants. This is addressed as the limitation, but should be also be interpreted in regards to difference in study results with Curhan *et al*. Despite the lower number of participants in the present study, the participants were well controlled age and gender. However, the youngest age group is inappropriately large. Dramatic differences in hearing are expected between the ages of 15-50 and should not be included in the same group.

 The definition of tinnitus in the text is incorrect. The authors reference a previous studies classification used in that research paradigm (performed in 1988), but is not generally accepted definition of tinnitus. This likely has resulted in the inaccurately inflated occurrence of tinnitus in used participants. Typically, tinnitus can be present in ~15-20% of the general public, but the ~63% reported in the control group in the present study is much higher than expected.

 Overall, the findings of the study could potentially be of interest. However, significant changes need to be made with clarity of writing, appropriate citations, divulging specific raw data to include frequency specific air and bone conduction results, better classification of tinnitus and labeling of such in raw data, along with other methodological changes to specifically address the question at hand.

I have read this submission. I believe that I have an appropriate level of expertise to confirm that it is of an acceptable scientific standard, however I have significant reservations, as outlined above.

# Response

**Published:** 2017-04-25

**Authors:** Apar Pokharel

**Affiliations:** College of Medical Sciences, Nepal

Thank you for reviewing my article.

 I want to add few explanations for your review comments.

 1. I have included noise induced hearing loss as my exclusion criteria. For ototoxic medications, I ha *ve taken detailed *history before recruiting the study population and have tried to exclude such patients.

 2. PTA was done for both ears.  

 3. DPOAE was not done this facility is not available in my institution.

 4. In my study the percentage of tinnitus has come higher, because both in my study population and control, I have included patients with comorbidities like DM, HTN which themselves are responsible for causing hearing loss.

 Thanks for your review.
